# Draft genomes of “*Pectobacterium peruviense*” strains isolated from fresh water in France

**DOI:** 10.1186/s40793-018-0332-0

**Published:** 2018-10-12

**Authors:** Pierre Faye, Claire Bertrand, Jacques Pédron, Marie-Anne Barny

**Affiliations:** Institute of Ecology and Environmental sciences-Paris, Sorbonne Université, INRA, 4 place Jussieu, F-75 252 Paris, France

**Keywords:** *Pectobacterium peruviense*, Soft rot, Plant pathogen, Water, France

## Abstract

Bacteria belonging to the genus *Pectobacterium* are responsible for soft rot disease on a wide range of cultivated crops. The “*Pectobacterium peruviense*” specie, recently proposed inside the *Pectobacterium* genus, gathers strains isolated from potato tubers cultivated in Peru at high altitude. Here we report the draft genome sequence of two strains belonging to “*P. peruviense*” isolated from river water in France indicating that the geographic distribution of this specie is likely to be larger than previously anticipated. We compared these genomes with the one published from the “*P. peruviense*” specie type strain isolated in Peru.

**Electronic supplementary material:**

The online version of this article (10.1186/s40793-018-0332-0) contains supplementary material, which is available to authorized users.

## Introduction

The *Pectobacterium* genus [1] gathers important plant pathogens that cause soft rot disease on a large variety of plant species [2]. Given their ability to cause disease on major crops, such as potato, *Pectobacterium* sp. have mainly been isolated from diseased plant during initial outbreak or sustained epidemic and their descriptions outside of agricultural context is scarce [3].

The classification of the *Pectobacterium* genus has been subject to extensive revision over the last decade. It is currently subdivided in 7 species; *P. carotovorum* [1], *P. atrosepticum* [4], *P. betavasculorum* [4], *P. wasabiae* [4]*,* *P. aroidearum* [5] *P. polaris* [6], *P. parmentieri* [7] and the recently proposed “*P. peruviense”* [8]. The *P. carotovorum* specie is heterogeneous and is currently subdivided several recognized subspecies, *P. carotovorum subsp. carotovorum* [9, 10], *P. carotovorum subsp. odoriferum* [9, 10] and proposed subspecies “*P. carotovorum* subsp. *actinidiae*” [11] and “*P. carotovorum subsp. brasiliense*” [12]. This heterogeneity led to assignation of many *Pectobacterium* isolates to *P. carotovorum**.* One example is the strain UG32 (also named IFB5232, SCRI179, LMG30269 and PCM2893) that was initially described as *P. carotovorum subsp. carotovorum* and is now the proposed type strain of the “*P. peruviense*” specie [8, 13]. All the strains described so far in the “*P. peruviense*” specie have been isolated in Peru in the seventies during the twentieth century from potato plants cultivated at high altitude (2400–3800 m). Here we described the draft genome sequence of two strains A97-S13-F16 and A350-S18-N16 isolated in February and November 2016 at different altitudes in the Durance river stream in France.

## Organism information

### Classification and features

Strain A97-S13-F16 was isolated in february 2016 from fresh water sampled in the river Durance while strain A350-S18-N16 was isolated in november 2016 from fresh water sampled in river Bléone, close to the confluent with river Durance. The fresh water parameters measured at the sampling times respectively were the following respectively for A97-S13-F16 and A350-S18-N16 sampled water: temperature 6.4 °C and 10.4 °C; turbidity 2.69 NTU and 145 NTU, conductivity 629 μS and 629 μS. Following sampling, 500 ml of fresh water was filtered through 0.2 μm pore filters (Sartorius cellulose acetate filters), the bacteria present on the filters were suspended in 1 ml sterile distilled water and 100 μl of the suspension were poured onto semi selective modified single-layers CVP_AG366_ plates (same medium as described in [14] except that tryptone was not added to the medium, hereafter described as CVP). After 2 days of growth at 28 °C, two strains forming pits on CVP medium were further isolated, named A97-S13-F16 and A350-S18-N16 and stored in 40% /60% glycerol/ LB liquid medium (10 g tryptone, 5 g yeast extract, 10 g NaCl per one liter of medium) at − 80 °C.

**Fig. 1:**
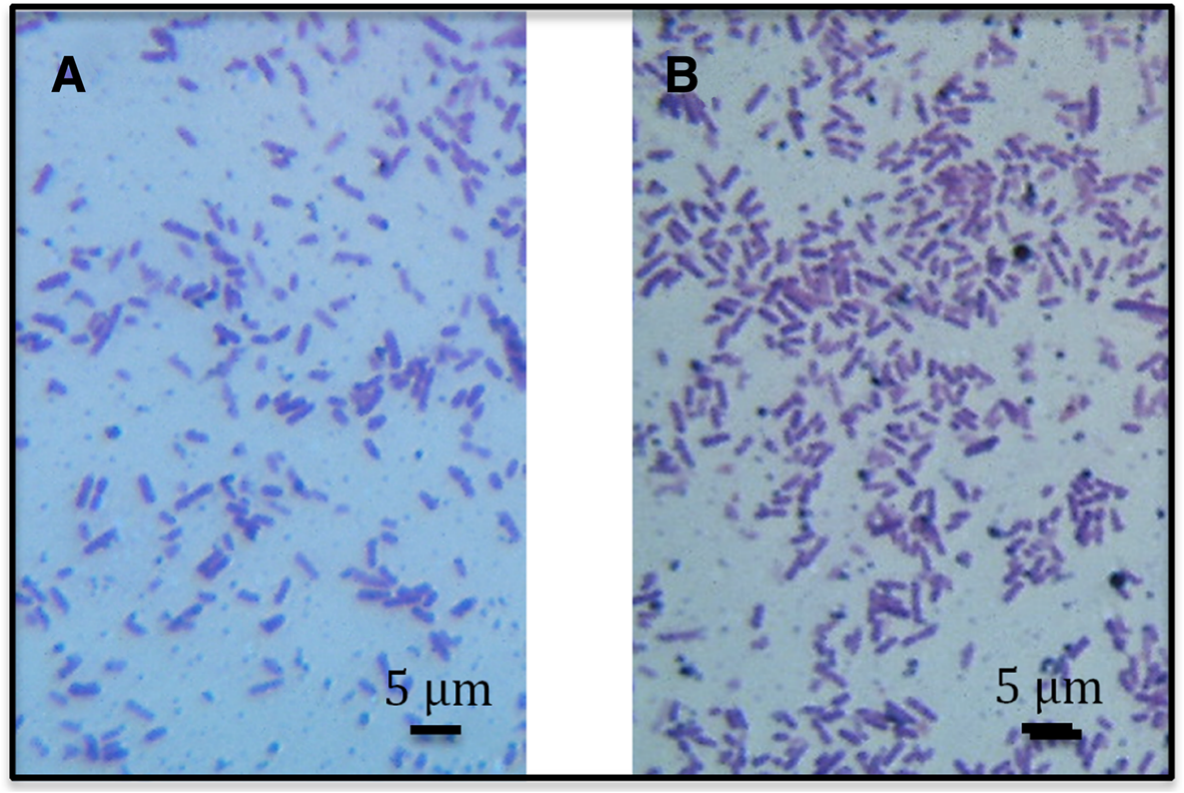
Photomicrographs of Gram stained exponentially growing “*P. peruviense*” cells. (**a**) strain A97-S13-F16, (**b**) A350-S18-N16. A light microscope with 100X magnification was used. These photomicrographs show the rod shaped forms of both strains. The bar scale represent 5 μm

Cells of both strains are rod shaped with length of approximately 2 μm in the exponential growth phase on LB medium (Fig. 1) and both strains are macerating potato tubers (Additional file 1: Figure S1). They are forming isolated colonies after 24 h at 28 °C on LB-15 g agar medium and after 48 h at 28 °C on TSA 10% medium (1,5 g tryptone, 0,5 g soy peptone, 0,5 g NaCl, 15 g agar per one liter of medium) and are inducing pits in CVP medium after 48 h at 28 °C.

**Fig. 2:**
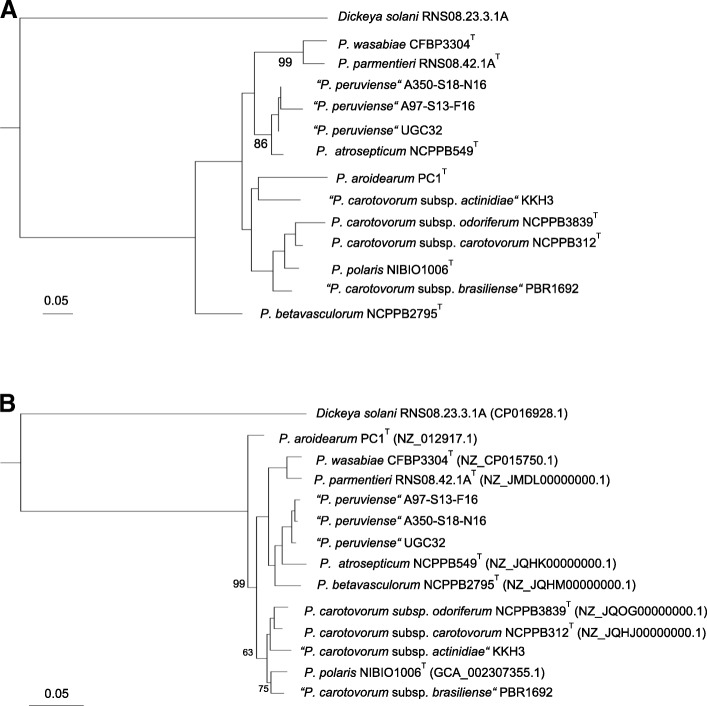
Phylogenetic trees of “*P. peruviense*” strains and strains of other *Pectobacterium* species and subspecies. **a** Phylogenetic tree constructed from the *gap*A nucleotide sequences. Sequences were aligned using the MUSCLE software [24] and the alignments were filtered by using the program GBLOCKS [25].Tree was computed using PHYML [26]. One hundred bootstrap replicates were performed to assess the statistical support of each node. Bootstrap support values (percentages) are indicated if superior to 95%. *gap*A sequences were retrieved from full genome of type strains (accession numbers are indicated in Fig. 1b) or obtained from the sequenced *gap*A amplicon for strains A97-S13-F16 and A350-S18-N16. **b** Phylogenetic tree constructed from concatenated sequences of 1266 homologous amino acid sequences. Before concatenation, the homologous sequences of each gene were aligned using the MUSCLE software [24] and the alignments were filtered by using the program GBLOCKS [25]. Tree was computed using PHYML [26]. One hundred bootstrap replicates were performed to assess the statistical support of each node. Bootstrap support values (percentages) are shown if less than 100%. The accession number for each genome is indicated inside brackets after the strain name. *Dickeya solani* RNS08.23.3.1.A was used as outgroup. Type strains are marked with T after the strain name

Amplification and sequencing of the *gap*A house keeping gene was recently described to rapidly characterize the different *Pectobacterium* species [15]. The *gap*A sequences of strains A97-S13-F16 and A350-S18-N16 clustered with the one of proposed “*P. peruviense*” type strain (Fig. 2A) and the clusterization of both strains with “*P. peruviense*” was confirmed through MLSA analysis of full genomes (Fig. 2B).

General feature of A97-S13-F16 and A350-S18-N16 are indicated in Table 1.

**Table 1:** Classification and general features of strains A97-S13-F16 and A350-S18-N16

MIGS ID	Property	Term	Evidence code^a^
	Classification	Domain *Bacteria*	TAS [14]
		Phylum *Proteobacteria*	TAS [15]
		Class *Gammaproteobacteria*	TAS [16]
		Order *Enterobacterale*	TAS [17]
		Family *Pectobacteriaceae*	TAS [17]
		Genus *Pectobacterium*	TAS [18]
		Species *Pectobacterium peruviense*	NAS
		strains: *A97-S13-F16 and A350-S18-N16*	
	Gram stain	*Negative*	NAS
	Cell shape	*Rod*	IDA
	Motility	*Motile*	IDA
	Sporulation	*Non sporulating*	NAS
	Temperature range	*Able to grow at 25 °C–28 °C*	IDA
	Optimum temperature	*Able to grow at 20 °C–30 °C*	NAS
	pH range; Optimum	*Unknown*	NAS
	Carbon source	*Ability to degrade polypectate*	IDA
MIGS-6	Habitat	*Isolated from water,*	IDA
MIGS-6.3	Salinity	*Able to grow in 1% NaCL*	IDA
MIGS-22	Oxygen requirement	*Facultative anaerobic*	NAS [17]
MIGS-15	Biotic relationship	*Free-living*	IDA
MIGS-14	Pathogenicity	*Ability to rot potato tubers*	IDA
MIGS-4	Geographic location	*A97-S13-F16, river Durance France*	IDA
		*A359-S18-N18, river Bléone France*	
MIGS-5	Sample collection	*A97-S13-F16, 1st February 2016*	IDA
		*A359-S18-N18, 19th November 2016*	
MIGS-4.1	Latitude	*A97-S13-F16, 44.701667*	IDA
		*A359-S18-N18, 44.038631*	
MIGS-4.2	Longitude	*A97-S13-F16, 6.599693*	IDA
		*A359-S18-N18, 5.967728*	
MIGS-4.4	Altitude	*A97-S13-F16, 907 m*	IDA
		*A359-S18-N18, 438 m*	

^a^ Evidence codes - *IDA* Inferred from Direct Assay, *TAS* Traceable Author Statement (i.e., a direct report exists in the literature), *NAS* Non-traceable Author Statement (i.e., not directly observed for the living, isolated sample, but based on a generally accepted property for the species, or anecdotal evidence). These evidence codes are from the Gene Ontology project [20]

## Genome sequencing information

### Genome project history

The aim of the project was to described *Pectobacterium* sp. isolated from environmental samples outside agricultural context. Fresh water sampling was performed in the river Durance and its tributaries in 2016. Amongst the isolated strains, the two strains A97-S13-F16 and A350-S18-N16, isolated in different locations and at different months in the river stream, were selected for sequencing following amplification and sequencing of their *gap*A house keeping gene because phylogenetic analysis of their *gap*A sequences positioned both *gap*A sequences close to the gapA sequence of the recently proposed “*P. peruviense*” type strain UGC32 [8, 13, 15].

### Growth conditions and DNA isolation

After isolation from fresh water in 2016, strains A97-S13-F16 and A350-S18-N16 have been stored in 40%/60% glycerol /LB medium at − 80 °C. For preparation of genomic DNA, the strains were first grown overnight at 28 °C on solid LB medium. A single colony was then pick up and grown overnight in 2 ml of liquid LB medium at 28 °C with 120 rpm shaking. Bacterial cells were harvested by centrifugation (5 min at 12,000 rpm) and DNA was extracted with the wizard® genomic DNA extraction kit (Promega) following the supplier specification. DNA was suspended in 100 μl of sterile distilled water and the quantity and quality of DNA was assessed by nano-drop measurement, spectrophotometry analysis and gel analysis.

### Genome sequencing and assembly

Genome sequencing was performed at the next generation sequencing core facilities of the Institute for Integrative Biology of the Cell, Bât. 21, Avenue de la Terrasse 91,190 Gif-sur-Yvette Cedex France. Nextera DNA libraries were prepared from 50 ng of high quality genomic DNA. Paired end 2 × 75 bp sequencing was performed on an Illumina NextSeq500 instrument, with a High Output 150 cycle kit.

CLC Genomics Workbench (Version 9.5.2, Qiagen Bioinformatics) was used to assemble 30,066,500 (mean length 53 bp) and 8,174,334 reads (mean length 52 bp) for strains A97-S13-F16 and A350-S18-N16 respectively. Final sequencing coverages were 331× and 86× with 61 and 73 scaffolds for strains A97-S13-F16 and A350-S18-N16 respectively (Table 2).

**Table 2:** Genome sequencing project information

MIGS ID	Property	*P. Peruviense*	*P. Peruviense*
		A97-S13-F16	A350-S18-N16
	Finishing quality	61 scaffolds	73 scaffolds
MIGS-28	Libraries used	Nextera DNA Library	Nextera DNA Library
MIGS 29	Sequencing platforms	Illumina NS500	Illumina NS500
MIGS 31.2	Fold coverage	331X	86X
MIGS 30	Assemblers	CLC Genomics	CLC Genomics
		Workbench V 9.5.2	Workbench V 9.5.2
MIGS 32	Gene calling method	Glimmer 3	Glimmer 3
	Locus Tag	A97-S13-F16	A350-S18-N16
	Genbank ID	PYUO01000000	PYUO0100000
	GenBank Date of Release	10th july 2018	10th july 2018
	GOLD ID		
	BIOPROJECT	PRJNA445781	PRJNA445781
MIGS 13	Source Material Identifier	CFBP8625 ^a^	CFBP8626 ^a^
	Project relevance	Environment	Environment

^a^ Strains A97-S13-F16 and A350-S18-N16 are available at the CIRM-CFBP Collection under the indicated numbers

### Genome annotation

Coding sequences were predicted using the RAST server [16] with the Glimmer 3 prediction tool [17]. COG assignments and Pfam domain predictions were done using the Web CD-Search Tool [18]. CRISPRFinder [19] was used to detect CRISPRs. Signal peptide and transmembrane domain were detected with the SignalP 4.1 Server [20] and transmembrane helices were predicted with TMHMM [21].

## Genomes properties

The “*P. peruviense*” A97-S13-F16 draft genome contains 4,775,191 bp with a GC content of 51%. Total predicted genes are 4503 while predicted protein coding genes are 4459 and RNA genes 44. The final assembly comprised 61 scaffolds. Among the predicted genes, 72.21% have a predicted function, 79.91% were assigned to COG and 85.40% have a predicted Pfam domain. Among the predicted proteins, 392 have a predicted signal peptide while 1090 contain a predicted transmembrane helix. Three CRIPS repeats array were detected in this genome.

The “*P. peruviense*” A350-S18-N16 draft genome contains 4,871,019 bp with a GC content of 51,1%. Total predicted genes are 4635 while predicted protein coding genes are 4487 and RNA genes 48. The final assembly comprised 73 scaffolds. Among the predicted genes, 72.01% have a predicted function, 78.77% were assigned to GOG and 85.09% have a predicted Pfam domain. Among the predicted proteins, 395 have a predicted signal peptide while 1095 contain a predicted transmembrane helix. Two CRIPS repeats array were detected in this genome.

The properties and the statistics of the two draft genomes are summarized in Tables 3 and 4.

**Table 3:** Genome statistics

Attribute	*P. peruviense* A97-S13-F16		*P. peruviense* A350-S18-N16	
	Value	% of Total	Value	% of Total
Genome size (pb)	4,755,191	100.00	4,871,019	100.00
DNA coding (bp)	4,108,775	86.41	4,211,847	86.47
DNA G + C (pb)	2,425,147	51.00	2,489,091	51.10
DNA scaffolds	61		73	
Total genes	4503	100.00	4635	100.00
Protein coding genes	4459	99.02	4587	98.96
RNA genes	44	0.97	48	1.03
Pseudo genes	NA		NA	
Genes in internal clusters	NA		NA	
Genes with function prediction	3252	72.21	3338	72.01
Genes assigned to COGs	3563	79.91	3613	78.77
Genes with Pfam domains	3808	85.40	3903	85.09
Genes with signal peptides	392	8.79	395	8.52
Genes with transmembrane helices	1090	24.44	1095	23.62
CRISPR repeats	3		2

**Table 4:** Number of genes associated with the 25 COG functional categories

Code	*P. peruviense*		*P. peruviense*		Description
	A97-S13-F16		A350-S18-N16		
	Value	%age	Value	%age	
E	366	8.21	367	8.00	Amino acid transport and metabolism
G	362	8.12	356	7.76	Carbohydrate transport and metabolism
D	41	0.92	43	0.94	Cell cycle control, cell division, chromosome partitioning
N	110	2.47	107	2.33	Cell motility
M	239	5.36	244	5.32	Cell wall/membrane/envelope biogenesis
H	167	3.75	167	3.64	Coenzyme transport and metabolism
Z	1	0.02	1	0.02	Cytoskeleton
V	85	1.91	90	1.96	Defense mechanisms
C	226	5.07	225	4.91	Energy production and conversion
W	4	0.09	4	0.09	Extracellular structures
S	202	4.53	209	4.56	Function unknown
G	204	4.58	206	4.49	General function prediction only
P	242	5.43	240	5.23	Inorganic ion transport and metabolism
U	82	1.84	91	1.98	Intracellular trafficking, secretion, and vesicular transport
I	103	2.31	102	2.20	Lipid transport and metabolism
X	23	0.52	57	1.24	Mobilome: prophages, transposons
F	90	2.02	91	1.98	Nucleotide transport and metabolism
O	152	3.41	152	3.31	Posttranslational modification, protein turnover, chaperones
L	127	2.85	127	2.77	Replication, recombination and repair
A	1	0.02	1	0.02	RNA processing and modification
Q	59	1.32	59	1.29	Secondary metabolites biosynthesis, transport and catabolism
T	146	3.27	148	3.23	Signal transduction mechanisms
K	291	6.53	287	6.26	Transcription
J	238	5.34	239	5.21	Translation, ribosomal structure and biogenesis
–	898	20.14	974	21.23	Not in COGs

The total %age is based on the total number of protein coding genes in the genome

## Insight from genome sequences

### Genome comparison between A97-S13-F16 and A350-S18-N16 and the genome of representative species of the *Pectobacterium* genus

A phylogenetic tree, constructed from concatenated sequences of 1266 homologs proteins, clustered the A97-S13-F16 and A350-S18-N16 strains together, close to UGC32 the proposed “*P. peruviense*” type strain (Fig. 1B). ANIb were further calculated between genomes of strains A97-S13-F16 and A350-S18-N16 and the genomes of described *Pectobacterium* species and subspecies (Additional file 2: Table S1). Pairwise ANIb values between the three “*P. peruviense*” genomes, A97-S13-F16 and A350-S18-N16 and UGC32, were above 97,5%. Pairewise ANIb values of these three “*P. peruviense*” genomes with genomes of other *Pectobacterium* species and subspecies were below 94%. dDDH is an in silico method to approach the wet-lab DDH method as closely as possible [22]. dDDH were calculated between the genomes of A97-S13-F16 and A350-S18-N16 and *Pectobacterium* genomes representative of known species and subspecies (Additional file 2: Table S1). dDDH values between A350-S18-N16, A97-S13-F16 genomes and the proposed “P. peruviense” UGC32 genomes were above 79%, well above the 70% species boundary. When pairwise calculations were performed between these three genomes with those of known *Pectobacterium* species and subspecies the estimated dDDH values dropped below 54%, well below the species boundary. This confirmed that A97-S13-F16 and A350-S18-N16 belong to the “*P. peruviense*” specie.

### Genomes comparison between the “*P. peruviense*” strains

**Fig. 3:**
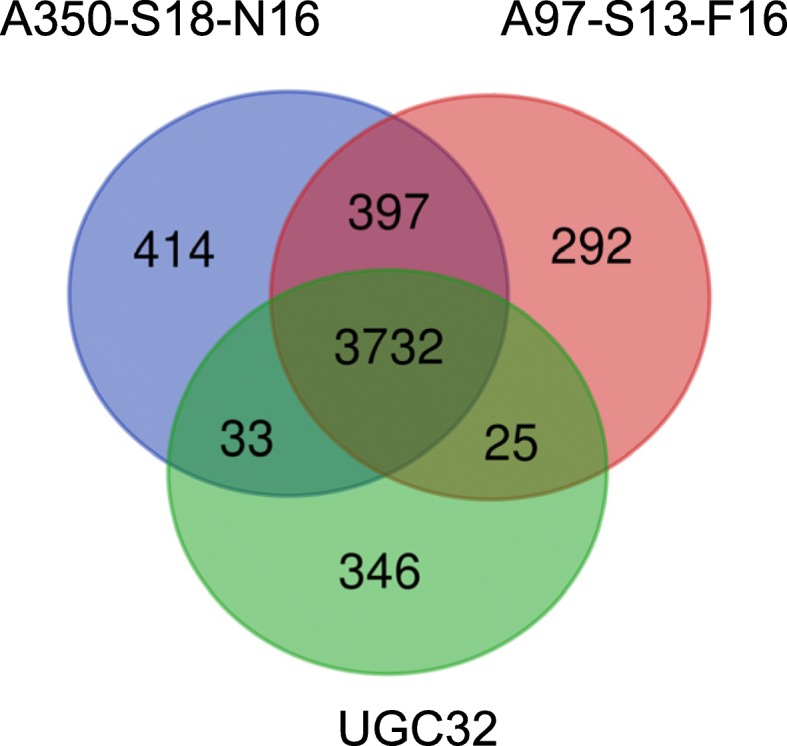
Venn diagram. Shared and unique genes between the genomes of “*P. peruviense*” A97-S13-F16 and A350-S18-N16 and the proposed “*P. peruviense*” type strain UGC32. Orthology was assumed using a threshold of 80% identity on at least 80% of the protein length

The phylogenetic trees (Fig. 2) indicate that strains A97-S13-F16 and A350-S18-N16 are more closely related to each other than they are from the “*P. peruviense*” type strain UGC32. To further gain insight into the distance between the three “*P. peruviense*” strains, we looked for shared and unique genes between genomes of strains A97-S13-F16, A350-S18-N16 and UGC32 type strain (Fig. 3). A97-S13-F16, A350-S18-N16 and UGC32 strains contain respectively a pool of specific genes of 292, 414 and 346. The slightly higher pool of specific genes observed in strain A350-S18-N16 could be partly related to its higher content of mobile genetic elements inserted in its genome as described in Table 4. Indeed, we observed 3 clusters of phage-related genes in strain A350-S18-N16, only one being also detected in strain A97-S13-F16. The Venn diagram indicated that 4129 genes are shared between strains A97-S13-F16 and A350-S18-N16 while only 3757 and 3765 genes are respectively shared between the type strain UGC32 and A97-S13-F16 / A350-S18-N16. This confirmed that A97-S13-F16 and A350-S18-N16 genomes are more closely related to each other than they are with the genome of the proposed type strain UGC32.

## Conclusions

In this study we presented the draft genome sequences of two strains of “*P. peruviense*” isolated from fresh water in river stream in France. The “*P. peruviense*” specie has recently been proposed and, until our study, the described strains belonging to the “*P. peruviense*” specie have all been isolated on potato tubers in the altiplano in Peru [8]. The presence of strains belonging to the “*P. peruviense*” specie in two independent environmental samples in France indicates that the geographic distribution of this specie is likely to be larger than previously anticipated. Both French strains are able to rot potato tubers like the proposed type strain UG32. The two French isolates are more closely related to each other than they are with the type strain UGC32. Whether this reflects the geographic provenance (France vs Peru) or the niche provenance (water vs diseased plants) is unknown.

## Additional files

Additional file 1: **FigureS1.** Symptoms observed on potato tubers. Overnight cultures of bacterial strains A97-S13-F16 and A350-S18-N16 were suspended in 50 mM phosphate buffer pH 6.8 and adjusted to 1.0 at OD_580nm_. Tubers of *S. tuberosum* var. charlotte were inoculated with 10 μl of the cell suspension and placed at room temperature on wet paper towel in a plastic box. Six days post-infection, tubers were cut in half and representative symptoms are shown: A: A97-S13-F16, B: A350-S18-N16, C: 50 mM phosphate buffer pH 6.8. (DOCX 9779 kb)

Additional file 2: **Table S1.** ANIb and dDDH pairwise values. dDDH and ANIb are respectively presented in the upper and lower part of the matrix triangle. Strains belonging to the same species are highlighted in red. Specific threshold value is 96% for ANIb and 70% for DDH. ANIb values were computed using the Blast algorithm of the Jspecies package [23]. dDDH were calculated according to [22]. (DOCX 79 kb)

## Abbreviations

ANI: Average Nucleotide Identity
COG: Clusters of Orthologous Groups
CRISPR: Clustered Regularly Interspaced Short Palindromic Repeats
dDDH: digital DNA-DNA hybridization
gapA: glyceraldehyde-3-phosphate dehydrogenase A
MLSA: Multi Locus Sequence Analysis
NTU: Normalized Turbidity Unit
sp.: specie
subsp.: subspecie
