# Supplementary figures and images for: Draft genomes of “Pectobacterium peruviense” strains isolated from fresh water in France
Source: Stand Genomic Sci. 2018 Oct 12;13:27. doi: 10.1186/s40793-018-0332-0 (PMC6186074; doi:10.1186/s40793-018-0332-0)

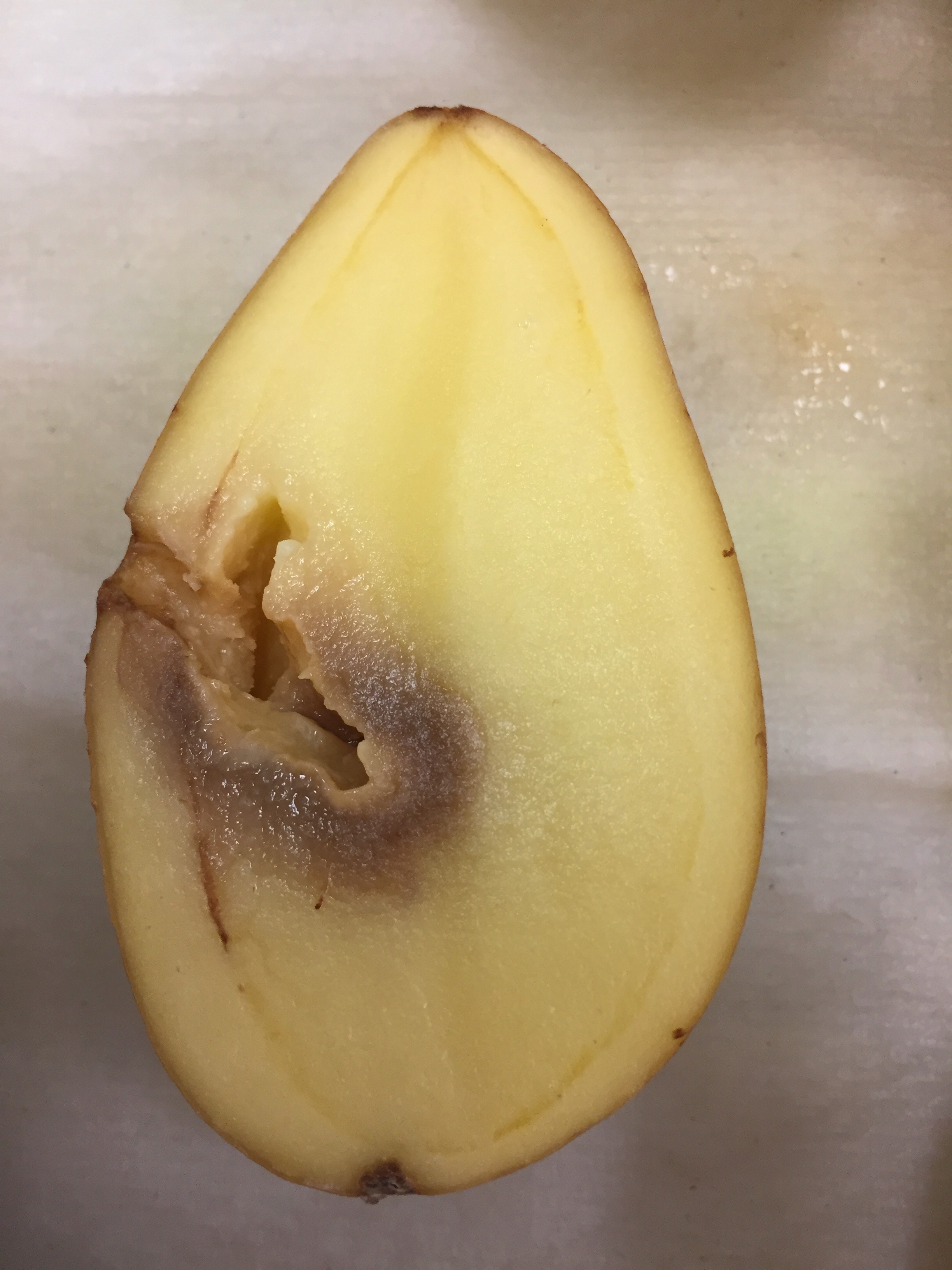

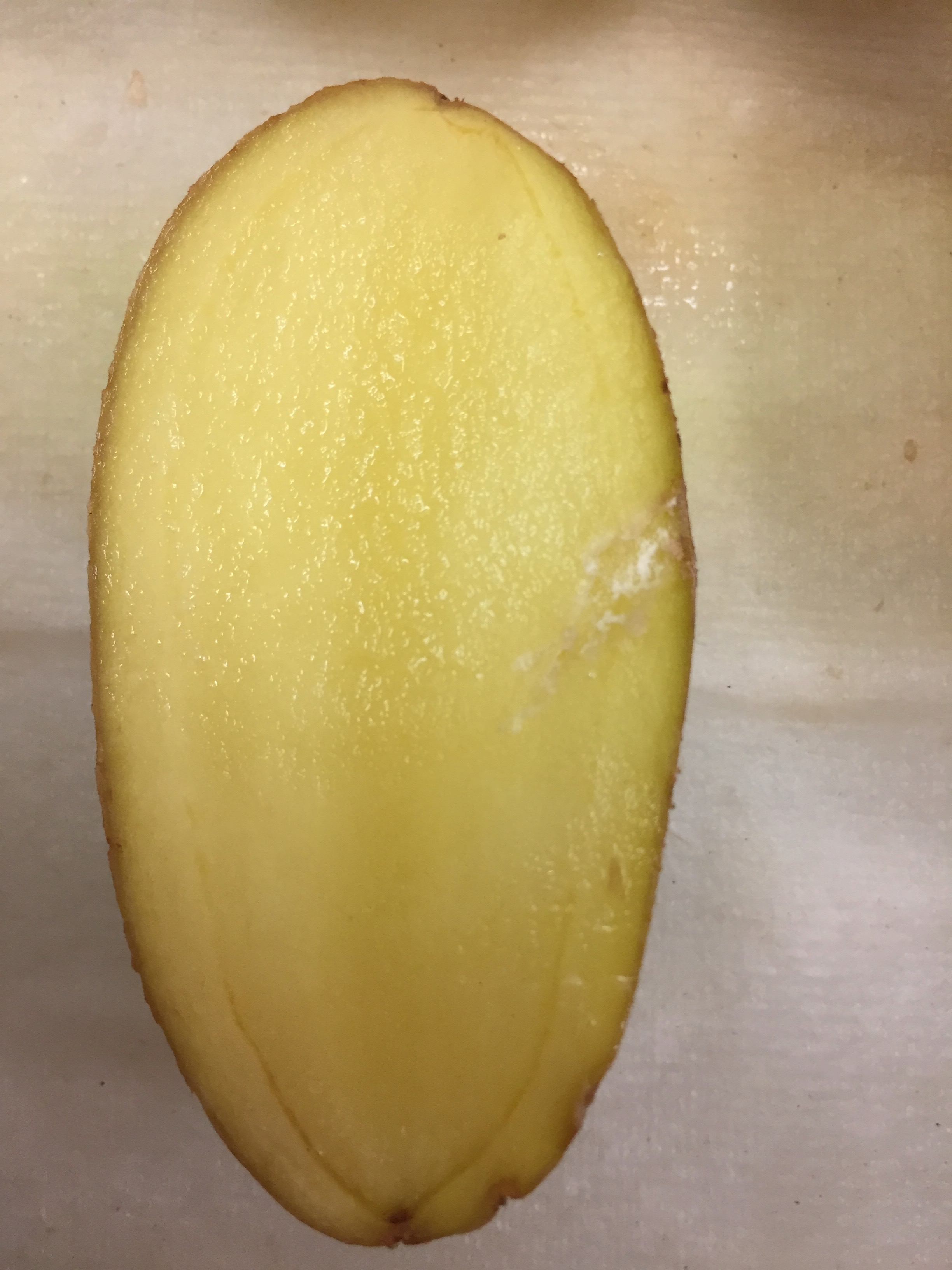

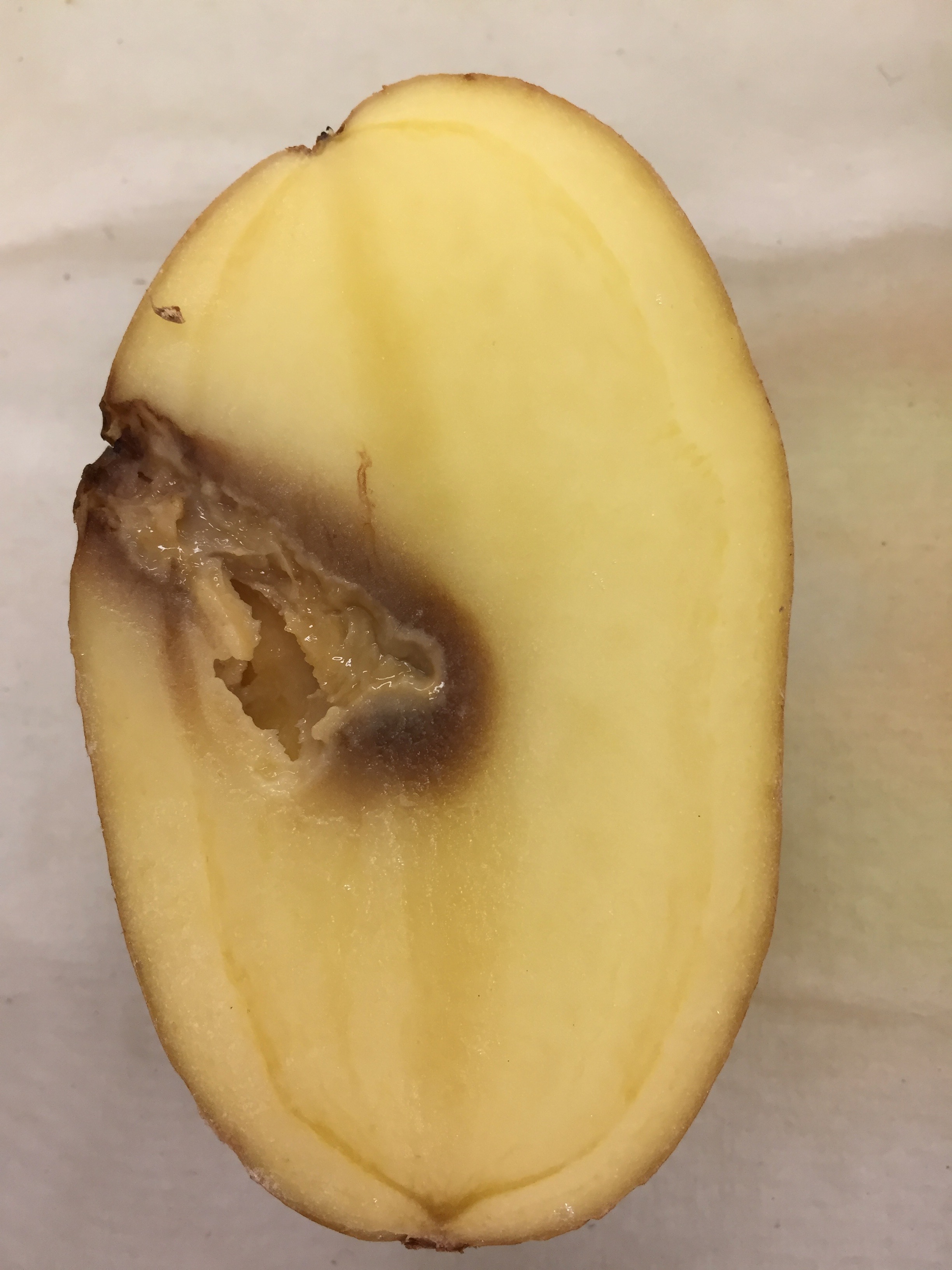


**A**

**B**

**C**

Supplement: Supplementary file 1 — FigureS1. Symptoms observed on potato tubers. Overnight cultures of bacterial strains A97-S13-F16 and A350-S18-N16 were suspended in 50 mM phosphate buffer pH 6.8 and adjusted to 1.0 at OD580nm. Tubers of S. tuberosum var. charlotte were inoculated with 10 μl of the cell suspension and placed at room temperature on wet paper towel in a plastic box. Six days post-infection, tubers were cut in half and representative symptoms are shown: A: A97-S13-F16, B: A350-S18-N16, C: 50 mM phosphate buffer pH 6.8. (DOCX 9779 kb) [file 40793_2018_332_MOESM1_ESM.docx]
