# Supplementary material for: Draft genomes of “Pectobacterium peruviense” strains isolated from fresh water in France
Source: Stand Genomic Sci. 2018 Oct 12;13:27. doi: 10.1186/s40793-018-0332-0 (PMC6186074; doi:10.1186/s40793-018-0332-0)
Supplement: Supplementary file 2 — Table S1. ANIb and dDDH pairwise values. dDDH and ANIb are respectively presented in the upper and lower part of the matrix triangle. Strains belonging to the same species are highlighted in red. Specific threshold value is 96% for ANIb and 70% for DDH. ANIb values were computed using the Blast algorithm of the Jspecies package [23]. dDDH were calculated according to [22]. (DOCX 79 kb) [file 40793_2018_332_MOESM2_ESM.docx]

|  | "*P. peruviense*" A350-S18-N16 | "*P. peruviense*" A97-S13-F16 | "*P. peruviense*" UGC32 | *P. atrosepticum* NCPPB549 | *P. betavasculorum* NCPPB2795 | *P. wasabiae* CFBP3304 | *P. parmentieri* RNS08.42.1A | *P. polaris* NIBIO1006 | *P. carotovorum subsp. carotovorum* NCPPB312 | *P. carotovorum subsp. odoriferum* NCPPB3839 | "*P. carotovorum subsp. brasiliense*" PBR1692 | "*P. carotovorum subsp. actinidiae*" KKH3 | *P. carotovorum aroidearum* PC1 |
| --- | --- | --- | --- | --- | --- | --- | --- | --- | --- | --- | --- | --- | --- |
| "*P. peruviense*" A350-S18-N16 | --- | 87.6 | 80.0 | 53.3 | 47.1 | 40.0 | 39.4 | 38.3 | 38.4 | 38.1 | 37.9 | 36.9 | 36.7 |
| "*P. peruviense*" A97-S13-F16 | 98.56 | --- | 79.4 | 53.6 | 47.2 | 40.0 | 39.3 | 38.2 | 38.3 | 37.9 | 37.9 | 36.9 | 36.5 |
| "*P. peruviense*" UGC32 | 97.68 | 97.59 | --- | 53.9 | 47.3 | 39.9 | 39.6 | 38.3 | 38.5 | 38.2 | 37.9 | 37.0 | 36.6 |
| *P. atrosepticum* NCPPB549 | 93.57 | 93.55 | 93.71 | --- | 46.8 | 40.0 | 39.4 | 38.5 | 38.8 | 38.3 | 38.1 | 37.4 | 36.6 |
| *P. betavasculorum* NCPPB2795 | 91.87 | 91.85 | 91.84 | 91.59 | --- | 38.5 | 37.8 | 38.3 | 38.1 | 37.7 | 38.1 | 36.8 | 36.8 |
| *P. wasabiae* CFBP3304 | 89.86 | 89.81 | 89.90 | 89.91 | 89.06 | --- | 54.7 | 36.8 | 36.8 | 36.5 | 36.4 | 35.8 | 35.6 |
| *P. parmentieri* RNS08.42.1A | 89.58 | 89.60 | 89.72 | 89.66 | 88.82 | 93.88 | --- | 36.6 | 36.7 | 36.5 | 36.3 | 35.6 | 35.7 |
| *P. polaris* NIBIO1006 | 89.40 | 89.37 | 89.35 | 89.53 | 89.26 | 88.75 | 88.57 | --- | 52.4 | 49.5 | 54.5 | 45.2 | 40.4 |
| *P. carotovorum subsp. carotovorum* NCPPB312 | 89.38 | 89.33 | 89.45 | 89.52 | 89.17 | 88.80 | 88.61 | 93.32 | --- | 61.5 | 51.0 | 51.0 | 40.3 |
| *P. carotovorum subsp. odoriferum* NCPPB3839 | 89.22 | 89.25 | 89.32 | 89.16 | 88.81 | 88.56 | 88.39 | 92.68 | 95.00 | --- | 47.4 | 50.1 | 39.5 |
| "*P. carotovorum subsp. brasiliense*" PBR1692 | 89.19 | 89.22 | 89.28 | 89.36 | 88.95 | 88.50 | 88.50 | 93.80 | 92.95 | 91.85 | --- | 43.9 | 41.3 |
| "*P. carotovorum subsp. actinidiae*" KKH3 | 88.80 | 88.83 | 88.84 | 88.99 | 88.45 | 88.19 | 87.99 | 91.55 | 92.98 | 92.47 | 91.04 | --- | 38.6 |
| *P. carotovorum aroidearum* PC1 | 88.73 | 88.71 | 88.73 | 88.76 | 88.60 | 88.20 | 88.18 | 90.11 | 90.04 | 89.68 | 90.33 | 89.32 | --- |
